# The Relative Cerebral Blood Volume (rCBV) < 42% Is Independently Associated with Collateral Status in Anterior Circulation Large Vessel Occlusion

**DOI:** 10.3390/jcm13061588

**Published:** 2024-03-10

**Authors:** Dhairya A. Lakhani, Aneri B. Balar, Manisha Koneru, Sijin Wen, Burak Berksu Ozkara, Hanzhang Lu, Richard Wang, Meisam Hoseinyazdi, Janet Mei, Risheng Xu, Mehreen Nabi, Ishan Mazumdar, Andrew Cho, Kevin Chen, Sadra Sepehri, Nathan Hyson, Victor Urrutia, Licia Luna, Argye E. Hillis, Jeremy J. Heit, Greg W. Albers, Ansaar T. Rai, Adam A. Dmytriw, Tobias Faizy, Max Wintermark, Kambiz Nael, Vivek S. Yedavalli

**Affiliations:** 1 Department of Radiology and Radiological Sciences, Johns Hopkins University, Baltimore, MD 21218, USA; aneribbalar@gmail.com (A.B.B.); hlu3@jhmi.edu (H.L.); rwang93@jhmi.edu (R.W.); mhosein3@jhmi.edu (M.H.); jmei12@jh.edu (J.M.); rxu4@jhmi.edu (R.X.); mnabi1@jhmi.edu (M.N.); imazumd1@jhmi.edu (I.M.); acho27@jhmi.edu (A.C.); kchen72@jhmi.edu (K.C.); ssepehr3@jh.edu (S.S.); nhyson1@jh.edu (N.H.); vurruti1@jhmi.edu (V.U.); lluna6@jhmi.edu (L.L.); vyedava1@jhmi.edu (V.S.Y.); 2 Cooper Medical School, Rowan University, Camden, NJ 08103, USA; 3 Department of Biostatistics, West Virginia University, Morgantown, WV 26506, USA; siwen@hsc.wvu.edu; 4 Department of Neurology, Johns Hopkins University, Baltimore, MD 21218, USAargye@jhmi.edu (A.E.H.); 5 Department of Neurology, Stanford University, Stanford, CA 94305, USA; jheit@stanford.edu (J.J.H.); albers@stanford.edu (G.W.A.); 6 Department of Neuroradiology, West Virginia University, Morgantown, WV 26506, USA; ansaar.rai@hsc.wvu.edu; 7 Department of Radiology, Harvarvd Medical School, Boston, MA 02115, USA; adam.dmytriw@gmail.com; 8 Department of Radiology, Neuroendovascular Division, University Medical Center Münster, 48149 Münster, Germany; tobiasfaizy@web.de; 9 Department of Neuroradiology, MD Anderson Medical Center, Houston, TX 77030, USA; max.wintermark@gmail.com; 10 Division of Neuroradiology, David Geffen School of Medicine, University of California Los Angeles, Los Angeles, CA 90095, USA; kambiznael@gmail.com

**Keywords:** relative cerebral blood volume, rCBV < 42%, ASITN collateral score

## Abstract

**Background:** The pretreatment CT perfusion (CTP) marker the relative cerebral blood volume (rCBV) < 42% lesion volume has recently been shown to predict 90-day functional outcomes; however, studies assessing correlations of the rCBV < 42% lesion volume with other outcomes remain sparse. Here, we aim to assess the relationship between the rCBV < 42% lesion volume and the reference standard digital subtraction angiography (DSA)-derived American Society of Interventional and Therapeutic Neuroradiology/Society of Interventional Radiology (ASITN) collateral score, hereby referred as the DSA CS. **Methods:** In this retrospective evaluation of our prospectively collected database, we included acute stroke patients triaged by multimodal CT imaging, including CT angiography and perfusion imaging, with confirmed anterior circulation large vessel occlusion between 1 September 2017 and 1 October 2023. Group differences were assessed using the Student’s *t* test, Mann–Whitney U test and Chi-Square test. Spearman’s rank correlation and logistic regression analyses were used to assess associations between rCBV < 42% and DSA CS. **Results:** In total, 222 patients (median age: 69 years, 56.3% female) met our inclusion criteria. In the multivariable logistic regression analysis, taking into account age, sex, race, hypertension, hyperlipidemia, diabetes, atrial fibrillation, prior stroke or transient ischemic attack, the admission National Institute of Health stroke scale, the premorbid modified Rankin score, the Alberta stroke program early CT score (ASPECTS), and segment occlusion, the rCBV < 42% lesion volume (adjusted OR: 0.98, *p* < 0.05) was independently associated with the DSA CS. **Conclusion:** The rCBV < 42% lesion volume is independently associated with the DSA CS.

## 1. Introduction

The cerebral blood volume (CBV) is an excellent quantitative marker for estimations of the collateral blood flow in the affected territory of acute ischemic stroke secondary to large vessel occlusion (AIS-LVO). There are a number of factors that impact the outcomes of AIS-LVOs, one of these is the robust collateral blood flow [referred to as collateral status (CS)] in the affected territories via pial arterial collateralization, hereby referred to as the collateral status [1,2,3,4,5,6,7,8]. A robust CS has been associated with better functional outcomes, a smaller infarct growth rate and better recanalization following mechanical thrombectomy (MT). Hence, CBV has the potential to be an excellent surrogate marker in estimations of CS and hence of functional outcomes. The CBV can be estimated via pretreatment CT perfusion (CTP) imaging [1,9,10,11]. Multiple different definitions have been used to quantify the CBV with varying success [6,12,13,14,15,16,17,18,19,20].

The majority of automated CTP software platforms have quantified the CBV relative to the non-affected or normal cerebral hemisphere [12,13,15,16,18,21]. The RAPID (IschemaView, Menlo Park, CA, USA) software platform quantifies the CBV using different thresholds of rCBV < 34%, rCBV < 38%, and rCBV < 42% in the region with a delayed contrast transit of Tmax > 6 s. These values are derived by dividing the average of all CBV values from Tmax > 6 s regions within the ischemic hemisphere by the average of all CBV values from unaffected brain tissue with a Tmax ≤ 4 s [21]. The RAPID-derived rCBV < 42% lesion volume has recently been shown to predict 90-day functional outcomes better than the rCBV < 34% and rCBV < 38% [22], likely attributable to the ability of the rCBV to quantify CS. 

Hence, in this study, we aim to assess the relationship of the rCBV < 42% lesion volume with the reference standard digital subtraction angiography (DSA)-derived American Society of Interventional and Therapeutic Neuroradiology/Society of Interventional Radiology (ASITN) collateral score, hereby referred to as the DSA CS, in anterior circulation in AIS-LVO patients. We hypothesize that increased rCBV < 42% volumes are associated with a poor DSA ASITN CS. 

## 2. Methods

### 2.1. Study Design

A retrospective analysis of prospectively maintained stroke databases was performed. Consecutive AIS-LVO patients from two comprehensive stroke centers were identified from 29 July 2019 to 1 October 2023. The Johns Hopkins University institutional review board (IRB # IRB00269637) approved this study. The strengthening the reporting of observational studies in epidemiology (STROBE) checklist guidelines for an observational study were used.

### 2.2. Study Participants

The inclusion criteria for this study were (a) Acute ischemic stroke secondary to an anterior circulation LVO confirmed on CTA images. LVO was defined as occlusion of the distal supraclinoid internal carotid artery (ICA) segment, middle cerebral artery (MCA) M1, and proximal MCA M2 segment. (b) Patients with a reported DSA ASITN CS. (c) Diagnostically adequate CT perfusion rCBV maps.

This study was performed in accordance with the Health Insurance Portability and Accountability Act (HIPAA) and the Declaration of Helsinki. Informed consent was waived by the IRB given the retrospective study design. The management decisions to administer thrombolysis or to perform interventions were made on an individual basis per institutional protocol based on a consensus stroke team evaluation.

### 2.3. Data Collection

The data were prospectively collected, and the database is actively managed. The baseline and clinical data were prospectively collected through electronic records, including, but not limited to, baseline demographics, clinical presentation, markers of stroke diagnosis and management, markers on baseline and follow-up imaging, various time parameters, and short- and long-term follow-up data.

### 2.4. CTP Image Acquisition

A Siemens Somatom Force (Erlangen, Germany) scanner was used. The acquisition parameters were 70 kVP, 200 Effective mAs, a rotation time of 0.25 s, an average acquisition time of 60 s, collimation of 48 × 1.2 mm, a pitch value of 0.7, and a 4D range of 114 mm × 1.5 s. 

### 2.5. Image Analysis

A quality assessment of CT perfusion data was performed independent of the outcomes by a board-certified neuroradiologist with 9 years of working experience. 

Commercial RAPID perfusion software, version 5.2.2 (iSchemaView, Menlo Park, CA, USA), was used to generate the rCBV < 42% lesion volume from raw CTP data.

The DSA ASITN CS was independently assessed by a board-certified neuroradiologist (9 years of working experience) and the performing neurointerventionalist. Any discrepancies were resolved based on a consensus review.

ASITN grades included Grade 0, non-existing or barely visible pial collaterals on the ischemic site at any point in time; Grade 1, partial collateralization of the ischemic site until the late venous phase; Grade 2, partial collateralization of the ischemic site by the late venous phase; Grade 3, complete collateralization of the ischemic site by the late venous phase; and Grade 4, complete collateralization of the ischemic site before the venous phase [6,23].

### 2.6. Statistical Analysis

The categorical data were described using contingency tables including counts and percentages; continuous variables were summarized with means (±standard deviation). A Student’s *t*-test was used in the data analysis for continuous variables, a Mann–Whitney U test was used in the data analysis for ordinal data, and a Chi-Square test was used for categorical data to assess differences in the variables in patients with a DSA ASITN CS of 0–2 (poor CS) and in those with an ASITN score of 3–4 (a good CS).

A logistic regression analysis was used to estimate the relationship between rCBV < 42% lesion volume and the dichotomized DSA ASITN CS as an outcome measure. The multivariable logistic regression model took into account confounding variables of age, sex, race, hypertension, hyperlipidemia, diabetes mellitus, atrial fibrillation, occlusion segment, prior transient ischemic attack or stroke, intravenous tissue-type plasminogen activator (IV tPA), the admission NIH stroke scale, the premorbid modified Rankin score (mRS), and the Alberta stroke program early CT score (ASPECTS). The outcomes were reported as adjusted and unadjusted odds ratios with a 95% confidence interval and a *P*-value. Statistically significant results are described as *p* ≤ 0.05, *p* < 0.01 and *p* < 0.001.

## 3. Results

A total of 222 consecutive patients, 125 (56.3%) female and 97 (43.7%) male, met our inclusion criteria. The mean age of the study cohort was 67.8 ± 15.8 years. A total of 116 (52.3%) Caucasian, 90 (40.5%) African American, 7 (3.2%) Asian, and 9 (4.1%) others were included.

In total, 174 (78.4%) had hypertension, 119 (53.6%) had hyperlipidemia, 58 (26.1%) had diabetes, 119 (53.6%) had heart disease and 44 (19.8%) had a prior history of stroke or transient ischemic attacks in our cohort. A total of 106 (48.6%) of the patient population had either a current or prior history of smoking.

Most of the patients in our cohort had a premorbid mRS of 0 (*n* = 147, 68.4%). Most of the patients in our cohort had an ASPECTS of 5 or more (*n* = 216). All six patients with an ASPECTS of 0–4 had a poor DSA ASITN CS, whereas using the rCBF < 30% threshold for infarct core estimation, a total of 36 patients (16.2%) met the definition of a large core (50 mL or greater).

In total, 80 patients (36%) received intravenous tissue plasminogen activator (IV tPA). The mean length of hospitalization was 11.6 days.

Of 222 patients, 161 (72.5%) had M1 segment occlusion, 45 (20.3%) had proximal M2 segment occlusion and 16 (7.2%) had distal ICA supraclinoid segment occlusion.

The patient demographics, imaging parameters and stroke treatment details are presented in Table 1.

### rCBV < 42% Lesion Volume and DSA ASITN CS

**Figure 1:**
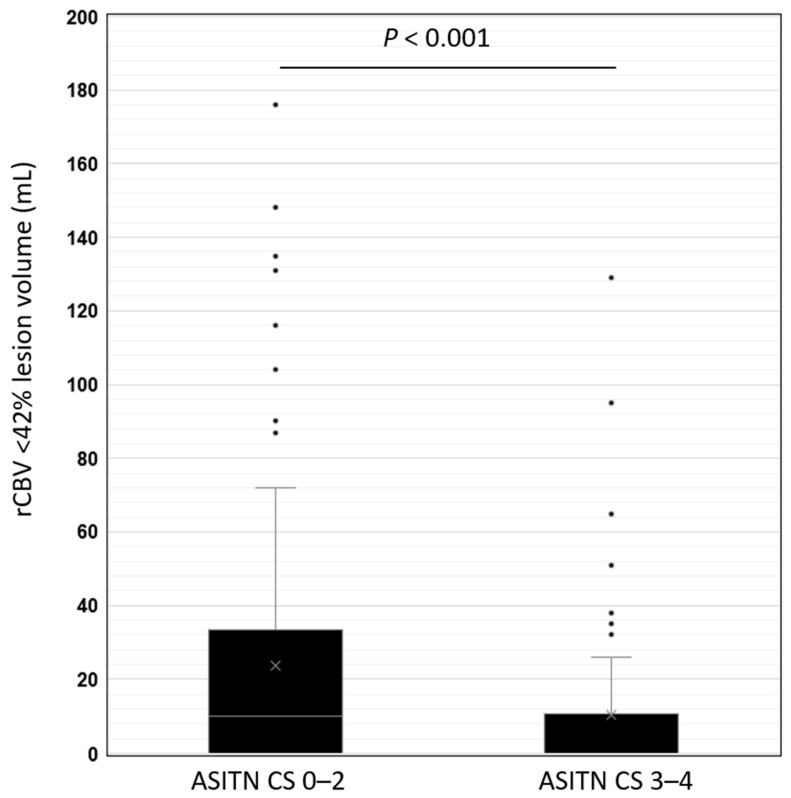
Box and whisker plot distribution of rCBV < 42% lesion volume in patients with a poor DSA ASITN CS of 0–2 and a robust DSA ASITN CS of 3–4. Box plot represents median and interquartile range, whereas whiskers represent minimum and maximum values.

The mean rCBV < 42% lesion volume was significantly higher in patients with a poor DSA ASITN CS (23.67 mL ± 34.85 mL), compared to a robust DSA ASITN CS (10.47 mL ± 21.69 mL, *p* < 0.001) (Figure 1 and Table 1).

In the logistic regression analysis, a lower rCBV < 42% lesion volume was associated with a good DSA CS (unadjusted OR: 0.98, 95% CI: 0.97–0.99, *p* < 0.01). Additionally, the admission NIHSS (unadjusted OR: 0.96, 95% CI: 0.92–0.99, *p* < 0.05) and segment occlusion (unadjusted OR: 1.35, 95% CI: 1.12–1.63, *p* < 0.001) were associated with the DSA ASITN CS (Table 2).

In the multivariable logistic regression analysis, which took into account age, sex, race, hypertension, hyperlipidemia, diabetes mellitus, atrial fibrillation, occlusion segment, prior transient ischemic attack or stroke, IV tPA, the admission NIH stroke scale, the premorbid mRS and the ASPECTS, the rCBV < 42% lesion volume (adjusted OR: 0.98, 95% CI: 0.97–0.99, *p* < 0.05) and segment occlusion (adjusted OR: 1.39, 95% CI: 1.13–1.72, *p* < 0.01) were independently associated with the DSA ASITN CS (Table 2).

## 4. Discussion

In this study of AIS-LVO patients undergoing MT triage, we found that a higher rCBV < 42% lesion volume was independently associated with a poor DSA ASITN CS. To our knowledge, the associations of different rCBV thresholds in estimating DSA ASITN CS remains sparse. This study validates an rCBV of <42% specifically as an additional prognostic biomarker of CS estimation in AIS-LVO patients.

The rCBV < 42% threshold lesion volume gives a quantitative estimate of the degree of hypoperfusion in the affected hemisphere. By definition, the rCBV quantifies the blood volume in the ischemic territory defined by delayed arterial transit of contrast, using a threshold of T max > 6 s, relative to the unaffected region defined by a normal arterial transit of contrast (T max ≤ 4 s). The threshold of a <42% flow relative to the unaffected region then provides the volume of the severely hyperperfused region in the affected territory, thereby serving as a quantitative marker of the CS [21,24].

Recently, the trial data from a review of the efficacy and safety of nerinetide for the treatment of acute ischemic stroke (ESCAPE-NA1 [22]) determined that an increased rCBV < 42% threshold lesion volume predicted functional outcomes at 90 days, as defined by the 90-day modified Rankin score (mRS). Moreover, in this study, the authors compared all three rCBV thresholds of rCBV < 34%, rCBV < 38%, and rCBV < 42%, and found that rCBV < 42% outperformed rCBV < 34% and rCBV < 38% lesion volumes in predicting 90-day functional outcomes [25]. It is postulated that rCBV thresholds estimate the degree of collateral blood flow withing the affected hemisphere and hence predict the functional outcome. However, there is only one study by Arenillas et al. [21] which studied the association of the rCBV with the DSA ASITN CS using SWIFT PRIME trial data. They report a significant association between rCBV and DSA CS (*p*  =  0.01). While the results of rCBV in predicting the DSA CS using SWIFT PRIME trial data validate rCBV as a reliable biomarker, patients with large cores were nevertheless excluded. Our study differs from that of Arenillas et al. in that we also include patients with large cores (≥50 mL) as these groups may have been underrepresented in their investigation [21]. Despite these differing inclusion criteria, our results nevertheless corroborate their observations. Our findings support the hypothesized physiological mechanism that a lower rCBV reflects a modest degree of compensation through poor collateral routes.

The main limitation of the reference standard DSA ASITN CS is that it is not available prior to intervention, and hence cannot be used in triaging patients for mechanical thrombectomies. Hence, there continues to be a growing interest in estimating the pre-treatment markers of CS. There are several markers which are already validated to estimate the CS, including those derived from single-phase CTA, multi-phase CTA, and CT perfusion [7]. Single-phase CTA CS markers include the Tan score [26] and the Clot Burden Score [27]. The Clot Burden Scoring system is a 10-point scoring system, where points are deducted based on vessel segments that are not opacified with contrast, whereas the Tan score system is a four-point grading system of the contrast opacification degree in the affected territory. The multiphase CTA-based mCTA scoring system is a six-point scoring system which takes into account dynamic filling of the collateral vessels during different contrast phases [28]. There are several CTP-based CS markers which are being validated, which include the Hypoperfusion Intensity Ratio (HIR), defined as the ratio of the Tmax >10 s lesion volume to the Tmax > 6 s lesion volume [8]; the CBV Index, defined as the relative CBV in the Tmax > 6 s region [8]; the compensation index, defined as the ratio of the Tmax > 4 s lesion volume to the Tmax > 6 s lesion volume [6]; and the relative cerebral blood flow (rCBF) < 38% lesion volume.

In conclusion, the rCBV < 42% lesion volume is independently associated with the reference standard DSA ASITN CS. Hence, it can be used as one of the potential pretreatment markers to estimate CS.

There are several limitations of this study to acknowledge. First, there were limitations that are inherent to a retrospective analysis. Second, there was modest inter- and intra-rater agreement in assessments of the DSA ASITN CS [29,30]. The strength of this study is the large sample size prospectively collected from two comprehensive stroke centers.

Our results validate the role of the rCBV < 42% lesion volume in estimating the DSA ASITN CS. More recently, large core trials showed the benefit of MTs in LVO cases irrespective of the ischemic core volume. Hence, the CTP-based rCBV < 42% lesion volume may be used as an adjunct prognostic marker in complex AIS-LVO cases. Future studies are needed to expand our understanding of the adjunct role of rCBV < 42% with other similar pretreatment imaging-based markers in clinical evaluation and decision making in AIS-LVO patients, particularly in the large-core subset of patients, those who did not undergo MT, and those with unsuccessful recanalization. 

## Figures and Tables

**Table 1:** Demographics of the study population, imaging biomarkers and treatment details.

	Poor ASITN CS of 0–2 (*n* = 150)		Good ASITN CS of 3 or 4 (*n* = 72)		Total (*n* = 222)		
	Number/Mean	%/Standard Deviation	Number/Mean	%/Standard Deviation	Number/Mean	%/Standard Deviation	*p* Value
Age	68.2	16.0	66.9	15.5	67.8	15.8	0.59
Sex							0.67
Female	83	55.3%	42	58.3%	125	56.3%	
Male	67	44.7%	30	41.7%	97	43.7%	
Race							0.27
African American	62	41.3%	28	38.9%	90	40.5%	
Caucasian	77	51.3%	39	54.2%	116	52.3%	
Asian	3	2.0%	4	5.6%	7	3.2%	
Other	8	5.3%	1	1.4%	9	4.1%	
Admission body mass index (kg/m^2^)	28.10	6.18	29.68	8.47	28.74	7.21	0.21
Hypertension	121	80.7%	53	73.6%	174	78.4%	0.23
Hyperlipidemia	79	52.7%	40	55.6%	119	53.6%	0.69
Diabetes Mellitus	38	25.3%	20	27.8%	58	26.1%	0.70
Heart Disease	79	52.7%	40	55.6%	119	53.6%	0.69
Atrial Fibrillation	56	37.3%	32	44.4%	88	39.6%	0.31
Smoking	70	47.6%	36	50.7%	106	48.6%	0.67
Prior transient ischemic attack/stroke	29	19.3%	15	20.8%	44	19.8%	0.79
Segment occlusion							<0.001
M1 segment	121	80.7%	40	55.6%	161	72.5%	
Proximal M2 segment	15	10.0%	30	41.7%	45	20.3%	
Supraclinoid ICA	14	9.3%	2	2.8%	16	7.2%	
Premorbid-modified Rankin score (mRS)							0.06
0	105	72.9%	42	59.2%	147	68.4%	
1	14	9.7%	12	16.9%	26	12.1%	
2	9	6.3%	8	11.3%	17	7.9%	
3	16	11.1%	7	9.9%	23	10.7%	
4	0	0.0%	2	2.8%	2	0.9%	
Admission NIH stroke scale	16	7	14	7	16	7	<0.05
Alberta stroke programme early CT score (ASPECTS)							0.10
0	2	1.3%	0	0.0%	2	0.9%	
3	2	1.3%	0	0.0%	2	0.9%	
4	2	1.3%	0	0.0%	2	0.9%	
5	8	5.3%	0	0.0%	8	3.6%	
6	11	7.3%	5	6.9%	16	7.2%	
7	11	7.3%	11	15.3%	22	9.9%	
8	25	16.7%	9	12.5%	34	15.3%	
9	24	16.0%	7	9.7%	31	14.0%	
10	65	43.3%	40	55.6%	105	47.3%	
rCBF <30% ≤ 50 ml	119	79.3%	67	93.1%	186	83.8%	<0.01
rCBV < 42% lesion volume (mL)	23.67	34.85	10.47	21.69	19.39	31.76	<0.001
Intravenous tissue-type plasminogen activator (IV tPA)	51	34.0%	29	40.3%	80	36.0%	0.36
Length of hospitalization	11.69	10.611	11.45	12.294	11.61	11.167	0.89
Last known well to door time in minutes	215.98	346.992	363.49	1258.901	269.21	801.792	0.39
Door to needle time in minutes	103.85	246.868	109.64	226.161	105.93	237.713	0.93
Door to groin puncture in minutes	195.76	86.337	172.85	131.386	187.49	104.525	0.38
Door to recanalization time in minutes	429.59	373.351	332.36	403.003	395.35	384.031	0.31

**Table 2:** Logistic regression analysis with a dichotomized poor DSA ASITN CS of 0–2 and a robust DSA CS of 3–4 as the outcome measure.

Variable	Univariable Analysis with Dichotomized Outcomes Based on a Good Collateral Score (ASITN 3 or More)				Multivariable Analysis with Dichotomized Outcomes Based on a Good Collateral Score (ASITN 3 or More)			
	Unadjusted OR	95% C.I.		*p* Value	Adjusted OR	95% C.I.		*p* Value
		Lower	Upper			Lower	Upper	
Age	1.00	0.98	1.01	0.59	0.99	0.97	1.01	0.38
Sex	0.88	0.50	1.56	0.67	0.92	0.48	1.78	0.81
Race	0.96	0.65	1.43	0.85	0.86	0.54	1.38	0.53
Hypertension	0.67	0.34	1.30	0.23	0.67	0.31	1.45	0.31
Hyperlipidemia	1.12	0.64	1.98	0.69	1.00	0.52	1.92	1.00
Diabetes Mellitus	1.13	0.60	2.14	0.70	0.99	0.46	2.11	0.98
Atrial Fibrillation	1.34	0.76	2.38	0.31	1.25	0.63	2.51	0.52
Prior stroke or Transient Ischemic Attack	1.10	0.55	2.21	0.79	1.26	0.57	2.77	0.57
Intravenous tissue-type plasminogen activator (IV tPA)	1.31	0.73	2.34	0.36	1.17	0.60	2.28	0.64
Admission NIH stroke scale	0.96	0.92	0.99	<0.05	0.96	0.92	1.01	0.16
Premorbid-modified Rankin score (mRS)	1.23	0.95	1.59	0.11	1.22	0.89	1.68	0.22
Alberta stroke programme early CT score (ASPECTS)	1.18	0.99	1.41	0.06	1.14	0.94	1.39	0.19
Segment occlusion	1.35	1.12	1.63	<0.001	1.39	1.13	1.72	<0.01
rCBV < 42% lesion volume	0.98	0.97	0.99	<0.01	0.98	0.97	0.99	<0.05

## Data Availability

The datasets used and/or analyzed during the current study are available from the corresponding author on reasonable request.

## Abbreviations

AIS-LVO: acute ischemic stroke secondary to large vessel occlusion; rCBV: relative CBV; MT: mechanical thrombectomy; NCCT: non-contrast CT head; CTA: CT angiography; CTP: CT perfusion; CS: collateral score; ICA: internal carotid artery; MCA: middle cerebral artery; IV tPA: intravenous tissue-type plasminogen activator (IV tPA); NIHSS: National Institute of Health stroke scale; mRS: modified Rankin score; ASPECTS: Alberta stroke program early CT score; mTiCI: modified treatment in cerebral infarction (mTICI) score.
